# Supplementary material for: Genome-wide probing of eukaryotic nascent RNA structure elucidates cotranscriptional folding and its antimutagenic effect
Source: Nat Commun. 2023 Sep 20;14:5853. doi: 10.1038/s41467-023-41550-w (PMC10511511; doi:10.1038/s41467-023-41550-w)
Supplement: Supplementary file 1 — Supplementary information [file 41467_2023_41550_MOESM1_ESM.pdf]

Supplementary Information for

Genome-wide probing of eukaryotic nascent RNA structure elucidates cotranscriptional folding and its antimutagenic effect

Gongwang Yu, Yao Liu, Zizhang Li, Shuyun Deng, Zhuoxing Wu, Xiaoyu Zhang, Wenbo Chen, Junnan Yang, Xiaoshu Chen, Jian-Rong Yang*

* Corresponding authors. E-mail: yangjianrong@mail.sysu.edu.cn (Yang, JR)

This PDF file includes:

Supplementary Figures 1 to 10
Supplementary Tables 1 and 2
References

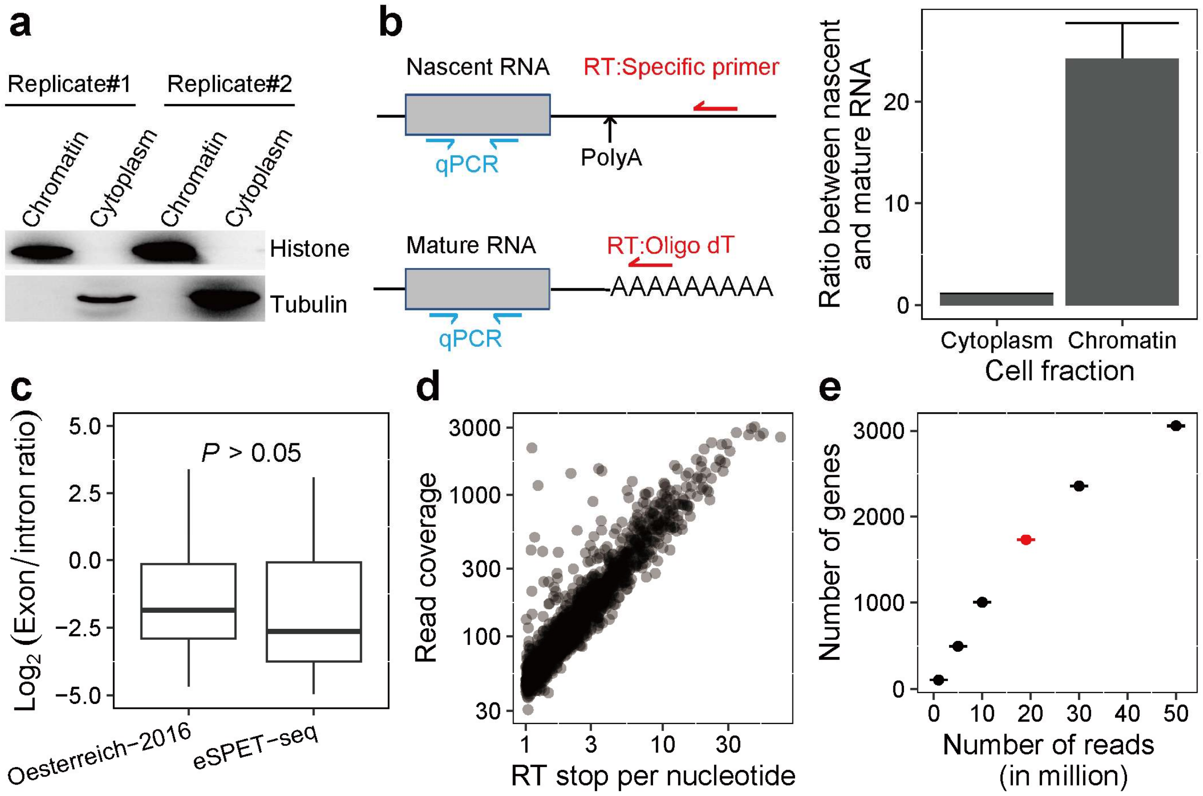

Supplementary Fig. 1: The eSPET-seq dataset provides rich and specific information about nascent RNA structure. a Western blot for histone H2b, a chromatin-associated protein, and tubulin, a cytoplasmic protein, in the chromatin fraction and the cytoplasmic fraction. The experiment was repeated twice with similar results. **b** The left panel describes the design of the primers. The right panel describes the results of qRT-PCR for the nascent (with the "RT:Specific" primer) and mature (with "RT: Oligo dT" primer) transcripts of *ADH1*. Data were presented as mean $\pm$ s.d. of three independent experiments. **c** For both datasets of Oesterreich-2016[1] and eSPET-seq, size-normalized ratios of reads mapping to exons versus introns were calculated (n = 44 genes per group). There was no significant difference between the two datasets, suggesting a similar level of enrichment for nascent RNA in the two studies. *P*-values are based on Wilcoxon signed-rank tests. Data are presented as standard box-and-whisker plots defined as in Fig.3. **d** Scatter plot of read coverage (y axis) versus RT stop per nucleotide (x axis) of a gene (n = 1601). The threshold of 1 RT stop per nucleotide corresponds to a read coverage of approximately 30. **e** The correspondence between sequencing depth and the number of genes with $\geqslant$ 1 RT stop per nucleotide. The red point indicates the estimated number using the full eSPET-seq dataset. Accordingly, the points to the left are inferred by down-sampling, while the points to the right are inferred by up-sampling. The error bars indicate standard deviation estimated from 100 re-sampling procedures. Source data are provided as a Source Data file.

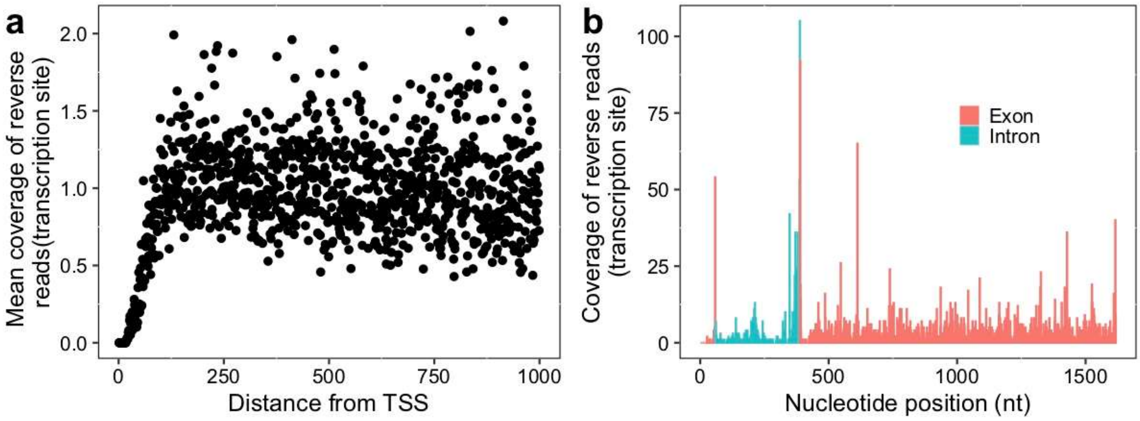

Supplementary Fig. 2: eSPET-seq captured transcription site. a Mean read density for the reverse reads from eSPET-seq, which indicate the transcription site, were averaged for the first 1,000 positions from the transcription start site (TSS) of all yeast genes. **b** Number of reverse reads from eSPET-seq at each position of an example gene (*YBR078W*). Note the similar coverage between exonic and intronic regions. Source data are provided as a Source Data file.

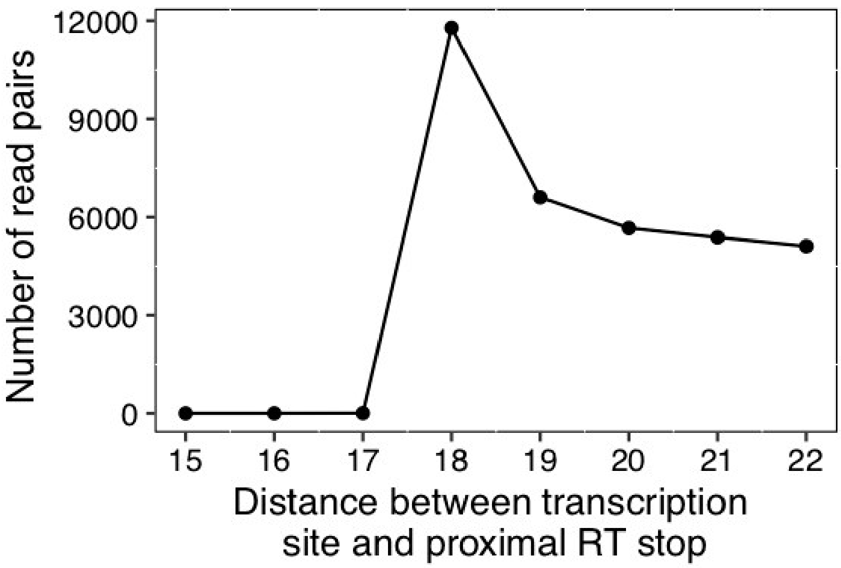

Supplementary Fig. 3: Number of nucleotides inaccessible by NAI-N_3 modification. The number of RT stops with a given distance to the corresponding transcription site was calculated for all transcriptional intermediates of all genes. The distance of 18 nt appeared as a peak, which was consistent with a previously reported number of nucleotides protected by the transcriptional elongation complex[2] and therefore cannot be modified by NAI-N_3. Source data are provided as a Source Data file.

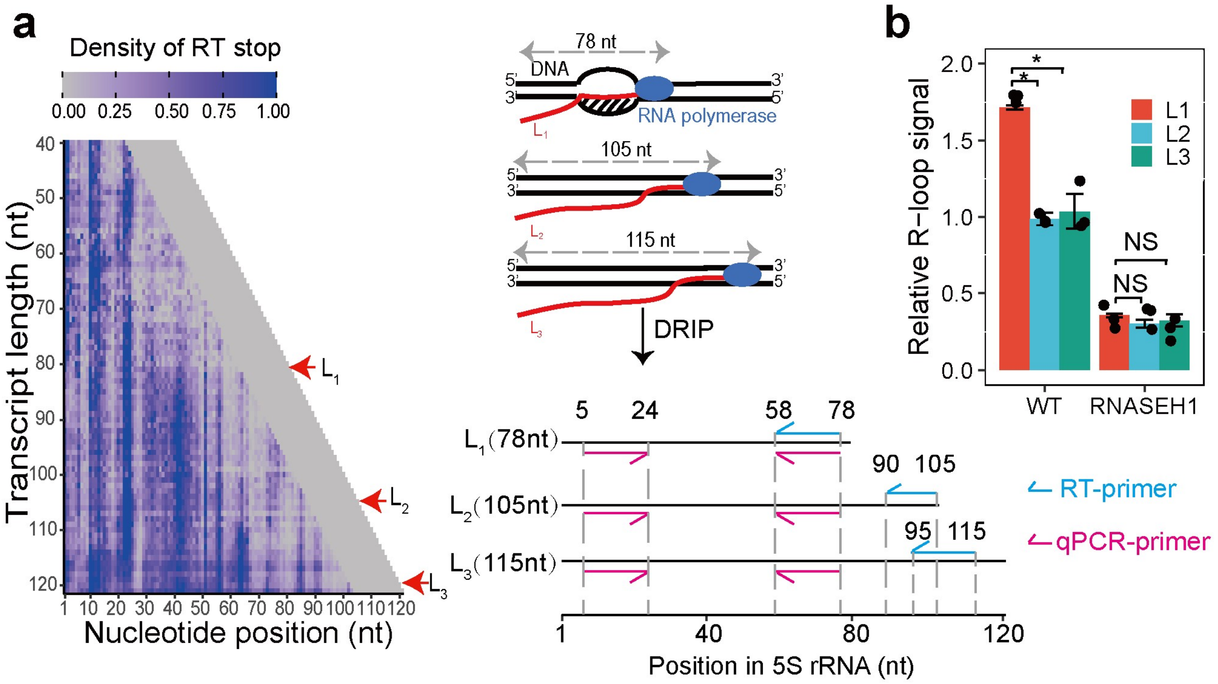

Supplementary Fig. 4: The structural transition in 5S rRNA was correlated with the dissolution of an R-loop. a Schematic diagram depicting the detection of the R-loop signal at nucleotides 30 to 45 of 5S rRNA. The density of RT stops in eSPET-seq data (same as shown in Fig. 2a) suggests a structural transition when transcription proceeds beyond the length of 80 nt. To confirm the corresponding R-loop dissolution,we performed DNA:RNA immunoprecipitation (DRIP) to enrich the R-loops, followed by reverse transcription using RT-primers designed for transcription intermediates L1 (length = 78 nt), L2 (105 nt) and L3 (115 nt), and finally qPCR using primers targeting nucleotides 30 to 45 to obtain a relative R-loop signal (relative to *ACT1*). **b** Wild-type strains (the "WT" group) showed a significantly higher R-loop signal in L1 than L2 and L3, consistent with R-loop dissolution for transcription beyond 80 nt. More importantly, when RNASEH1 (which hinders R-loop formation by degrading RNA) was overexpressed, all R-loop signals from L1, L2 and L3 decreased and their difference is no longer significant (the "RNASEH1" group), which suggests the previous significant difference is indeed R-loop-dependent. Together with the density of RT-stops from eSPET-seq, our results demonstrated the correlation between R-loop and structure transition for 5S rRNA. The results are represented as means ± s.d. of three independent experiments. *P*-values are based on a two-tailed t-test. $^*P < 0.01$; NS, non-significant. Source data are provided as a Source Data file.

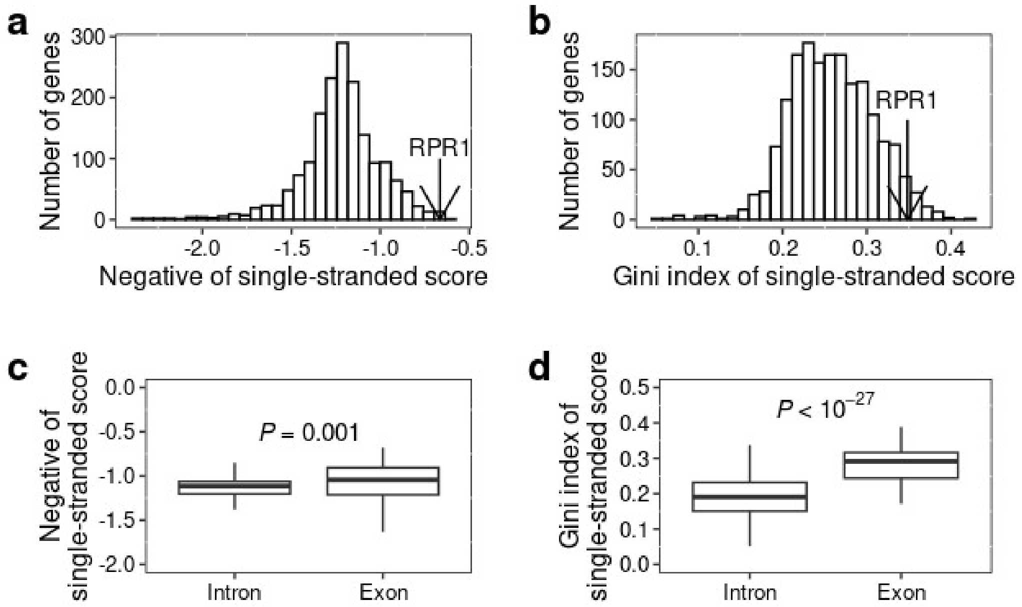

Supplementary Fig. 5: Prevalence of nascent RNA structure in yeast genes. a-b Prevalence of nascent RNA structure of each yeast gene was estimated by the negative single-stranded score (**a**) and Gini index of the single-stranded score (**b**). The distributions of both metrics were shown for 1,603 protein-coding genes and 125 noncoding genes with an average coverage of at least 1 read per nucleotide. According to both metrics, the nascent RNA folding strength ranged > 4-fold differences, suggesting substantial variations in the prevalence of nascent RNA folding. Among them, some protein-coding genes, for which RNA structures were generally believed unimportant for their main function of translation, have nascent RNA structures as prevalent as ncRNA (e.g., *RPR1*, encoding RNA component of nuclear RNase P, indicated by the arrow), for which the secondary structures are usually considered essential for their function. **c-d** Boxplot of negative of single-stranded score (**c**) and Gini index of single-stranded score (**d**) in exon versus in intron for intron-containing genes. *P*-values are based on Wilcoxon signed-rank tests. The exons showed stronger nascent RNA folding than the introns according to both metrics. Data are presented as standard box-and-whisker plots defined as in Fig.3. Source data are provided as a Source Data file.

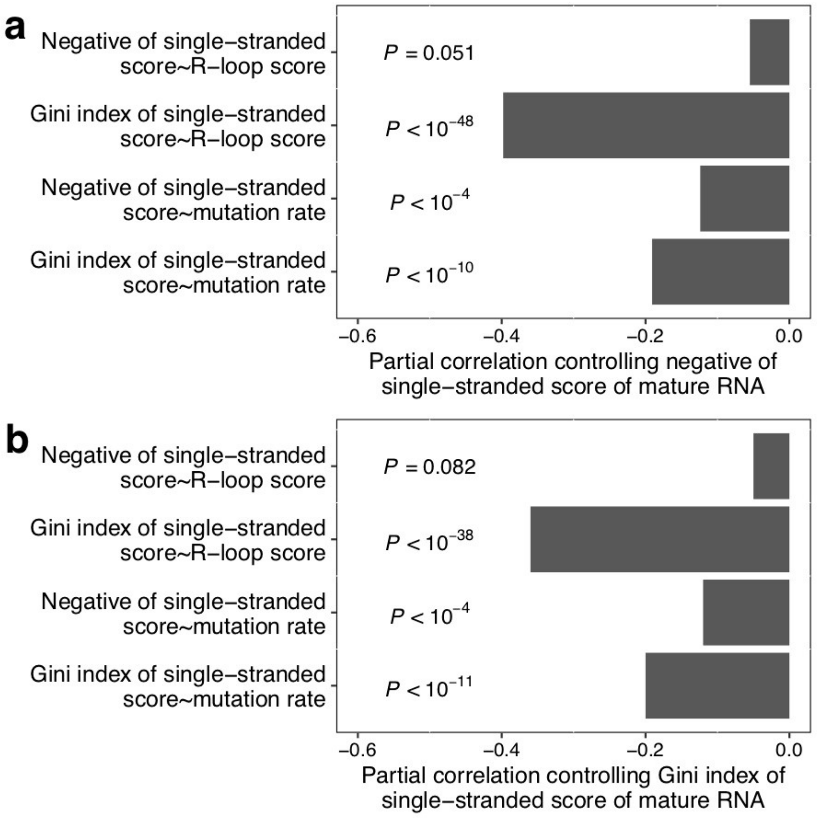

Supplementary Fig. 6: The correspondence among the nascent RNA folding, R-loop and mutation rate was not confounded by the mature RNA structure. For all correlations shown in Fig. 4b, c, e and f, partial correlations were calculated by controlling the prevalence of secondary structure in mature RNAs (see Methods) probed by icSHAPE. Negative of single-stranded score (**a**) or Gini index of single-stranded score (**b**) were respectively used to represent prevalence of secondary structure in mature RNAs. The insignificant minority, i.e., the correlation between the negative single-stranded score and R-loop score might be explained by the confounding effect of the number of reads hitting each gene.

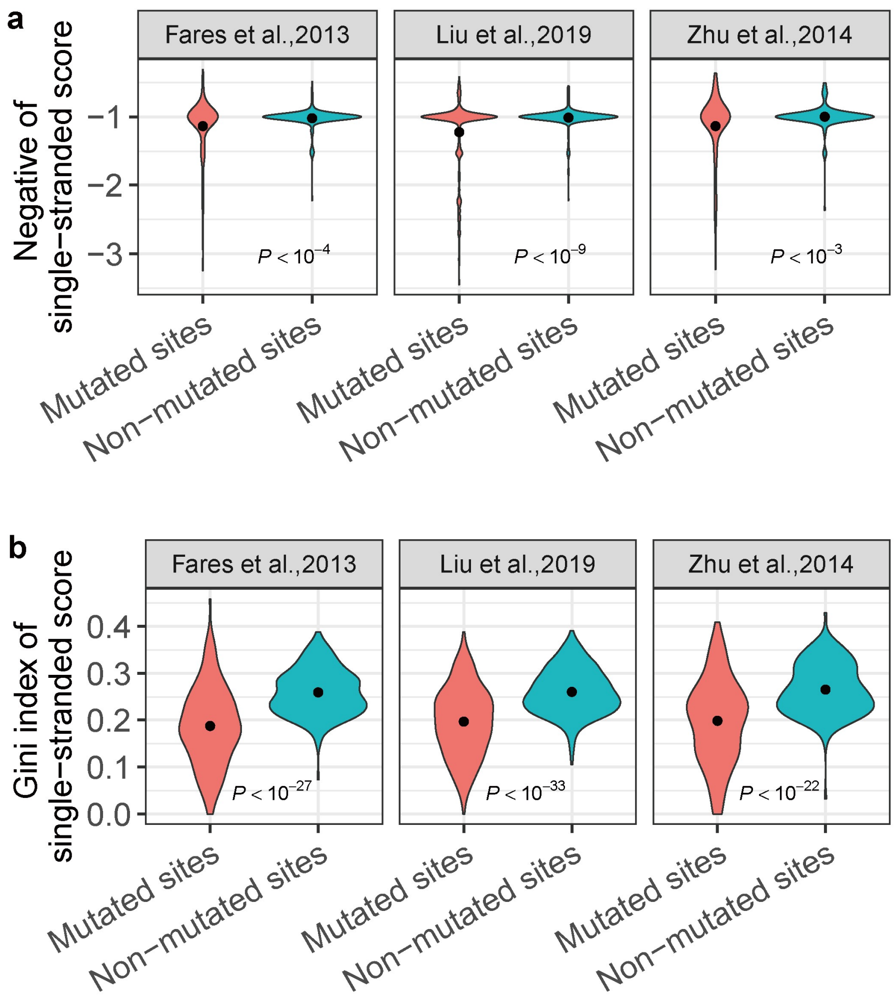

Supplementary Fig. 7: Mutated sites in mutation accumulation experiments[3-5] tend to have lower prevalence of nascent RNA structure compared to non-mutated sites in the same gene. **a-b** Violin plot for prevalence of nascent RNA secondary structure in mutated versus non-mutated sites in mutational accumulation experiments. The prevalence of nascent RNA secondary structure was approximated by either negative value (**a**) or Gini index (**b**) of single-stranded scores. The black dot inside the violin shows the mean. *P*-values are based on Wilcoxon signed-rank tests. Source data are provided as a Source Data file.

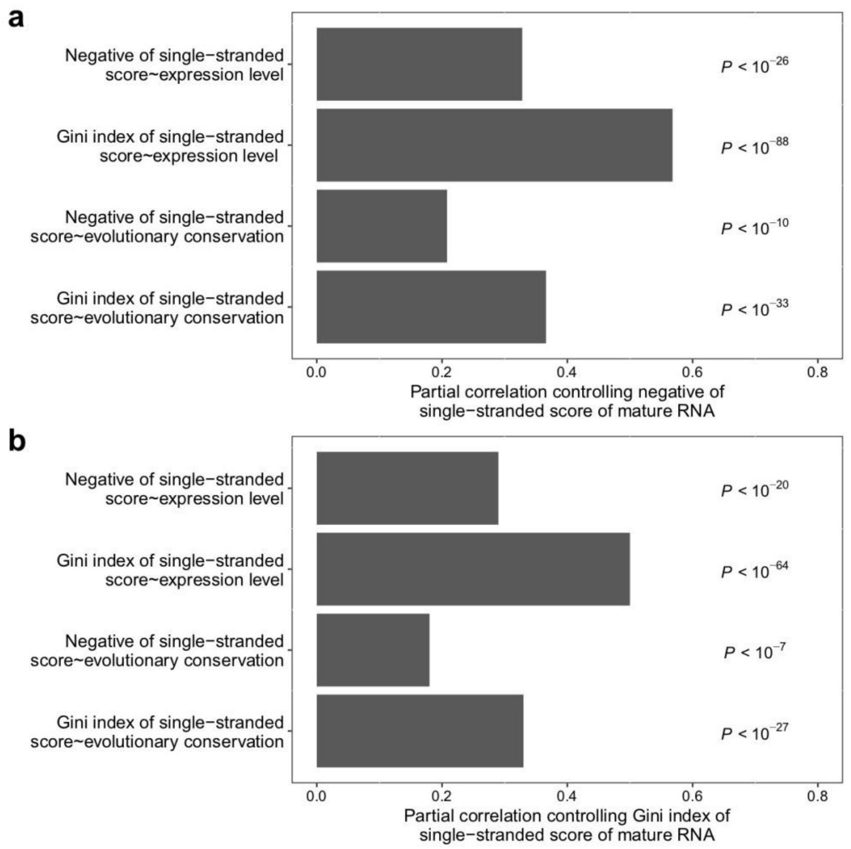

Supplementary Fig. 8: The strong nascent RNA folding of functionally constrained genes was not confounded by the structure of mature RNA. a-b For all correlations shown in Fig. 6a-d, partial correlations were calculated by controlling the prevalence of secondary structure in mature RNAs (see Methods) probed by icSHAPE. Negative of single-stranded score (**a**) or Gini index of single-stranded score (**b**) were respectively used to represent prevalence of secondary structure in mature RNAs.

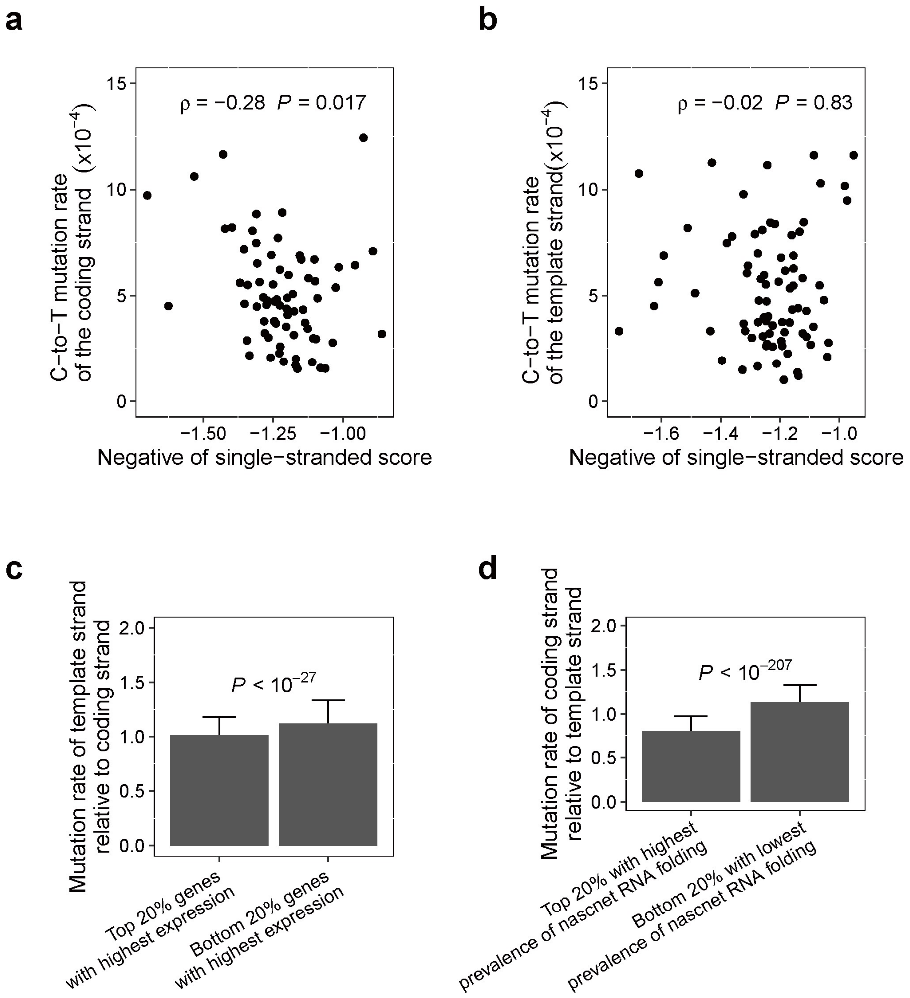

Supplementary Fig. 9: The antimutator effect of nascent RNA folding is independent of TCR. **a**-**b** Assuming dominate contribution by hydrolytic deamination of cytosine in C/G to T/A mutations[6], we estimated the mutation rate of C-to-T mutations on the coding (**a**) and the template (**b**) strand by pooling mutations detected in YPD-based mutation accumulation lines[3-5], and compared them with the prevalence of the nascent RNA structure (x axis). Spearman’ s rank correlation coefficients are indicated. (n = 267 for coding strand, n = 270 for template strand). **c** The C-to-T mutation rate of the template strand relative to the coding strand was compared between the top and bottom 20% genes with highest expression (and therefore TCR). The difference indicates the effect size of TCR. **d** The C-to-T mutation rate of the coding strand relative to the template strand was compared between the top and bottom 20% genes with highest prevalence of nascent RNA folding. The difference indicates the effect size of the antimutator effect by nascent RNA folding. Error bars represent 95% confidence intervals estimated by bootstrapping the genes 1000 times. P-values are based on Wilcoxon signed-rank tests. Source data are provided as a Source Data file.

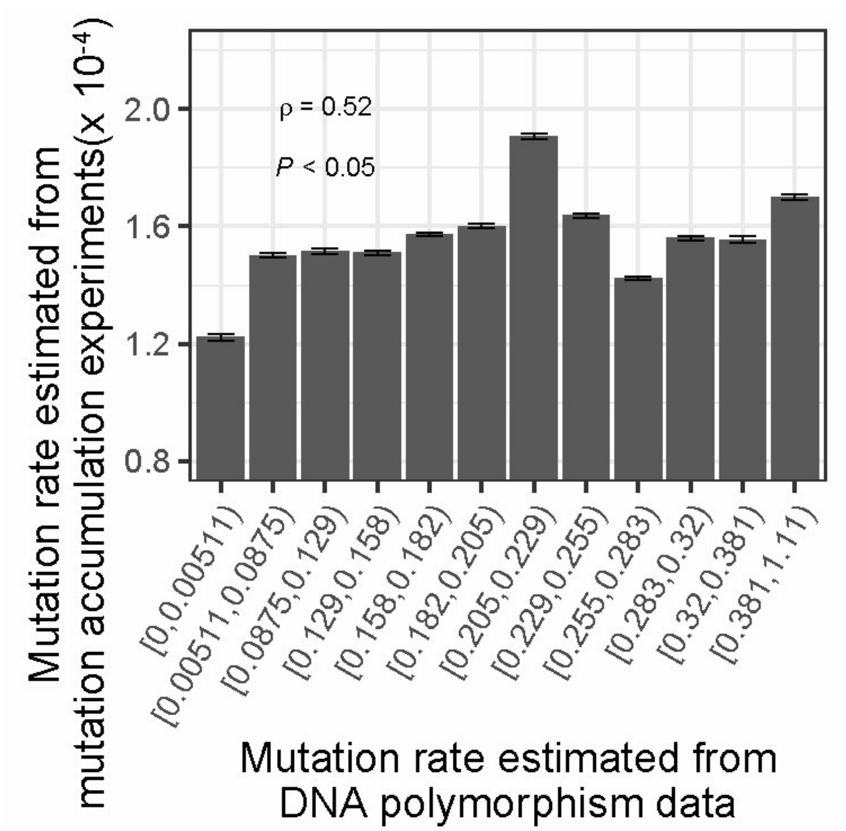

Supplementary Fig. 10: Correlation between the spontaneous mutation rate estimates derived from DNA polymorphisms of 190 *S. cerevisiae* strains and mutation accumulation experiments. The yeast genes with polymorphism-based mutation rate estimates are divided into 10 equal-sized groups with increasing mutation rate (*x* axis). The average mutation rate of each group, as estimated from the mutations detected in YPD-based mutation accumulation lines[3-5], was calculated (*y* axis) and found to be positively correlated with the polymorphism-based mutation rate. Error bars indicate the 95% confidence interval of the mean, estimated by bootstrapping the genes 1,000 times. Spearman's rank correlation and its significance (*P* value) is shown.

Supplementary Table 1: Summary statistics for each high-throughput sequencing library.

Experiment	Treatment	Biological replicates	Total number of read pairs	Number of uniquely mapped reads	Percentage of uniquely mapped reads
eSPET-seq	$NAI-N_3$ (in vivo)	Replicate #1	152459797	125635597	82.41%
		Replicate #2	192166325	168423947	87.64%
		Replicate #3	182110119	160368459	88.06%
	DMSO	Replicate #1	154255167	142550186	92.41%
		Replicate #2	105549641	98681537	93.49%
		Replicate #3	221399201	203362908	91.85%
icSHAPE	$NAI-N_3$ (in vivo)	Replicate #1	109091283	79291216	72.68%
		Replicate #2	119721437	79900718	66.74%
	$NAI-N_3$ (in vitro)	Replicate #1	102123344	81961244	80.26%
		Replicate #2	72339103	40301158	55.71%
	DMSO	Replicate #1	104058860	83987550	80.71%
		Replicate #2	363906727	230363355	63.30%
eSPET-seq targeting *CAN1*	-	-	18785686	10226571	54%

Supplementary Table 2: Primers used in this study.

Name	Sequence (5'- 3') *	Usage
3' biotinylated RNA adapter	/5rApp/AGATCGGAAGAGCACACGTC/<br>3Bio/	eSPET-seq library of nascent RNA
3' ddC RNA adapter	/5rApp/AGATCGGAAGAGCACACGTC/<br>3ddC/	eSPET-seq library of nascent RNA
RT primer	5'-GACGTGTGCTCTTCCGATCT-3'	eSPET-seq library of nascent RNA
single-stranded DNA adapter	/5Phos/NNNAGATCGGAAGAGCGTCG<br>TGTAG/3SpC3/	eSPET-seq library of nascent RNA
forward primer	5'AATGATACGGCGACCACCGAGATC<br>TACACTCTTTCCCTACACGACGCTCT<br>TCCGATCT-3'	eSPET-seq library of nascent RNA
reverse primer	5'CAAGCAGAAGACGGCATACGAGAT<br>NNNNNNGTGACTGGAGTTCAGACGT<br>GTGCTCTTCCGATC-3'	eSPET-seq library of nascent RNA
Reverse PCR primer targeting *CAN1* of weak version in NAI-N3 group	CAAGCAGAAGACGGCATACGAGATA<br>CATCGGTGACTGGAGTTCAGACGTG<br>TGCTCTTCCGATCT**TATGAGC**CATCT<br>TTCAAGGGTATCGATCC	eSPET-seq library targeting *CAN1*
Reverse PCR primer targeting *CAN1* of weak version in DMSO group	CAAGCAGAAGACGGCATACGAGATA<br>CATCGGTGACTGGAGTTCAGACGTG<br>TGCTCTTCCGATCT**ATTCCT**CATCTT<br>TCAAGGGTATCGATCC	Preparation of eSPET-seq library targeting *CAN1*
Reverse PCR primer targeting *CAN1* of intermediate version in NAI-N3 group	CAAGCAGAAGACGGCATACGAGATA<br>CATCGGTGACTGGAGTTCAGACGTG<br>TGCTCTTCCGATCT**ACAGTG**TCATC<br>TTTCAATGGTATTGACCC	Preparation of eSPET-seq library targeting *CAN1*
Reverse PCR primer targeting *CAN1* of intermediate version in DMSO group	CAAGCAGAAGACGGCATACGAGATA<br>CATCGGTGACTGGAGTTCAGACGTG<br>TGCTCTTCCGATCT**GCCAAT**TCATCT<br>TTCAATGGTATTGACCC	Preparation of eSPET-seq library targeting *CAN1*
Reverse PCR primer targeting *CAN1* of strong version in NAI-N3 group	CAAGCAGAAGACGGCATACGAGATA<br>CATCGGTGACTGGAGTTCAGACGTG<br>TGCTCTTCCGATCT**ATCACG**TCTTTC<br>AATGGTATAGAACCGC	Preparation of eSPET-seq library targeting *CAN1*
Reverse PCR primer targeting *CAN1* of strong version in DMSO group	CAAGCAGAAGACGGCATACGAGATA<br>CATCGGTGACTGGAGTTCAGACGTG<br>TGCTCTTCCGATCT**CGATGT**TCTTTC<br>AATGGTATAGAACCGC	Preparation of eSPET-seq library targeting *CAN1*
Forward PCR Primer	AATGATACGGCGACCACCGAGATCT<br>ACACTCTTTCCCTACACGACGCTCTT<br>CCGATCT	Preparation of eSPET-seq library targeting *CAN1*
RT primer for intermediate L1	CTCGGTCAGGCTCTTACCAG	DRIP-RT-qPCR

RT primer for intermediate L2	TTTCGCGTATGGTCA	DRIP-RT-qPCR
RT primer for intermediate L3	AGCACCTGAGTTTCGCGTAT	DRIP-RT-qPCR
Forward PCR primer for 5S rRNA	GCGGCCATATCTACCAGAAA	DRIP-RT-qPCR
Reverse PCR primer for 5S rRNA	AGGCTCTTACCAGCTTAACT	DRIP-RT-qPCR
RT primer for *CAN1*	GTCTGTGGTGCGTTTGCGACG	DRIP-RT-qPCR
Forward PCR primer for *CAN1*	GAAGACGCCGACATAGAGGA	DRIP-RT-qPCR
Reverse PCR primer for *CAN1*	GTCTGTGGTGCGTTTGCGACG	DRIP-RT-qPCR
RT primer for *ACT1*	random primer(N6)	DRIP-RT-qPCR
Forward PCR primer for *ACT1*	GGTGGTTCTATCTTGGCTTCTTTG	DRIP-RT-qPCR
Reverse PCR primer for *ACT1*	GATGGACCACTTTCGTCGTATTCT	DRIP-RT-qPCR
RT primer for *ADH1*	CCCAACTGAAGGCTAGGCTGTGG	Enrichment of *ADH1* nascent RNA
Forward PCR primer for *ADH1*	CGACGGTTCTTTCCAACAAT	Enrichment of *ADH1* nascent RNA
Reverse PCR primer for *ADH1*	ACGGTGATACCAGCACACAA	Enrichment of *ADH1* nascent RNA

*** :** The blue bold sequence is the barcode that is used to split each group of sequencing data. The underlined sequence is a specific primer targeting *CAN1*.

Supplementary References

1. Oesterreich, F.C. *et al.* Splicing of Nascent RNA Coincides with Intron Exit from RNA Polymerase II. *Cell* **165**, 372-381 (2016).
2. Fu, J. *et al.* Yeast RNA polymerase II at 5 A resolution. *Cell* **98**, 799-810 (1999).
3. Fares, M.A., Keane, O.M., Toft, C., Carretero-Paulet, L. & Jones, G.W. The roles of whole-genome and small-scale duplications in the functional specialization of Saccharomyces cerevisiae genes. *PLoS Genet* **9**, e1003176 (2013).
4. Zhu, Y.O., Siegal, M.L., Hall, D.W. & Petrov, D.A. Precise estimates of mutation rate and spectrum in yeast. *Proc Natl Acad Sci U S A* **111**, E2310-8 (2014).
5. Liu, H. & Zhang, J. Yeast Spontaneous Mutation Rate and Spectrum Vary with Environment. *Curr Biol* **29**, 1584-1591 e3 (2019).
6. Maki, H. Origins of spontaneous mutations: specificity and directionality of base-substitution, frameshift, and sequence-substitution mutageneses. *Annu Rev Genet* **36**, 279-303 (2002).
