# Supplementary material for: Genome-wide probing of eukaryotic nascent RNA structure elucidates cotranscriptional folding and its antimutagenic effect
Source: Nat Commun. 2023 Sep 20;14:5853. doi: 10.1038/s41467-023-41550-w (PMC10511511; doi:10.1038/s41467-023-41550-w)
Supplement: Supplementary file 3 — Reporting Summary [file 41467_2023_41550_MOESM3_ESM.pdf]

nature portfolio

Corresponding author(s): Jian-Rong Yang
Last updated by author(s): Aug 28, 2023

Reporting Summary

Nature Portfolio wishes to improve the reproducibility of the work that we publish. This form provides structure for consistency and transparency in reporting. For further information on Nature Portfolio policies, see our Editorial Policies and the Editorial Policy Checklist.

Statistics

For all statistical analyses, confirm that the following items are present in the figure legend, table legend, main text, or Methods section.

n/a	Confirmed	
☐	☒	The exact sample size (*n*) for each experimental group/condition, given as a discrete number and unit of measurement
☐	☒	A statement on whether measurements were taken from distinct samples or whether the same sample was measured repeatedly
☐	☒	The statistical test(s) used AND whether they are one- or two-sided<br>*Only common tests should be described solely by name; describe more complex techniques in the Methods section.*
☒	☐	A description of all covariates tested
☐	☒	A description of any assumptions or corrections, such as tests of normality and adjustment for multiple comparisons
☐	☒	A full description of the statistical parameters including central tendency (e.g. means) or other basic estimates (e.g. regression coefficient) AND variation (e.g. standard deviation) or associated estimates of uncertainty (e.g. confidence intervals)
☐	☒	For null hypothesis testing, the test statistic (e.g. *F*, *t*, *r*) with confidence intervals, effect sizes, degrees of freedom and *P* value noted<br>*Give P values as exact values whenever suitable.*
☒	☐	For Bayesian analysis, information on the choice of priors and Markov chain Monte Carlo settings
☒	☐	For hierarchical and complex designs, identification of the appropriate level for tests and full reporting of outcomes
☐	☒	Estimates of effect sizes (e.g. Cohen's *d*, Pearson's *r*), indicating how they were calculated

Our web collection on statistics for biologists contains articles on many of the points above.

Software and code

Policy information about availability of computer code

Data collection	Raw sequencing data were directly obtained from sequencing service providers, without specific softwares invovled.
Data analysis	All codes used in data analyses was available on https://github.com/GongwangYu/NascentRNAfolding_NS-seq.We have explicitly mentioned all software used during data analysis in the "Method" section, including Trimmomatic, cutadapt, Bowtie2.

For manuscripts utilizing custom algorithms or software that are central to the research but not yet described in published literature, software must be made available to editors and reviewers. We strongly encourage code deposition in a community repository (e.g. GitHub). See the Nature Portfolio guidelines for submitting code & software for further information.

Data

Policy information about availability of data

All manuscripts must include a data availability statement. This statement should provide the following information, where applicable:

- Accession codes, unique identifiers, or web links for publicly available datasets
- A description of any restrictions on data availability
- For clinical datasets or third party data, please ensure that the statement adheres to our policy

The new data generated in this study, i.e., eSPET-seq data, were deposited to NCBI Sequence Read Archive under accession number SRP291653. All other HTS data were extracted from previous publications, whose sources were all explicitly listed in the main text. Source data are provided with this paper.

Field-specific reporting

Please select the one below that is the best fit for your research. If you are not sure, read the appropriate sections before making your selection.

☒ Life sciences ☐ Behavioural & social sciences ☐ Ecological, evolutionary & environmental sciences

For a reference copy of the document with all sections, see nature.com/documents/nr-reporting-summary-flat.pdf

Life sciences study design

All studies must disclose on these points even when the disclosure is negative.

Sample size	Sample sizes of experiments was determined by the previous related studies on structure probing. And the determined samples sizes used in this study was acceptable and adequate for statistical tests.
Data exclusions	Data exclusions applied during the data analyses were detailed in the "Methods" section. Briefly, the transcript was excluded for analysis if it has a mean RT stop count lower than 1.
Replication	Each experiment was reproduced at least twice with similar results. All attempts at replication were successful.
Randomization	Randomization was not used in this study since all yeast strains are identical.
Blinding	Data analyses were conducted with identical pipeline for each experiment, therefore blinding is irrelevant here.

Reporting for specific materials, systems and methods

We require information from authors about some types of materials, experimental systems and methods used in many studies. Here, indicate whether each material, system or method listed is relevant to your study. If you are not sure if a list item applies to your research, read the appropriate section before selecting a response.

Materials & experimental systems

n/a	Involved in the study	
☐	☒	Antibodies
☒	☐	Eukaryotic cell lines
☒	☐	Palaeontology and archaeology
☒	☐	Animals and other organisms
☒	☐	Human research participants
☒	☐	Clinical data
☒	☐	Dual use research of concern

Methods

n/a	Involved in the study	
☒	☐	ChIP-seq
☒	☐	Flow cytometry
☒	☐	MRI-based neuroimaging

Antibodies

Antibodies used	anti-histone H2B,(Abcam,ab188291); anti-tubulin(b185224, Abcam)
Validation	All antibodies are commercially available and were commercially validated
